# Supplementary material for: Synthesis and Properties of Norphthalocyanines Functionalized with a Tetrathiacrown Ether–Tetrathiafulvalene Substituent
Source: Molecules. 2023 Jan 17;28(3):916. doi: 10.3390/molecules28030916 (PMC9920445; doi:10.3390/molecules28030916)
Supplement: Supplementary file 1 [file molecules-28-00916-s001.zip › molecules-2113563-supplementary.pdf]

Supplementary Data

Synthesis and properties of norphthalocyanines functionalized with a tetrathiacrown ether–tetrathiafulvalene substituent

Ruibin Hou [1,2], Xiaoyu Liu [1,2], Yan Xia [1,2]* and Dongfeng Li [1,*]

[1] School of Chemistry and Life Science, Changchun University of Technology, Changchun, 130012, China

[2] Advanced Institute of Materials Science, Changchun University of Technology, Changchun 130012, China

* Correspondence: hmz20130521@163.com (Y.X.); lidongfeng@mail.ccut.edu.cn (D.L.)

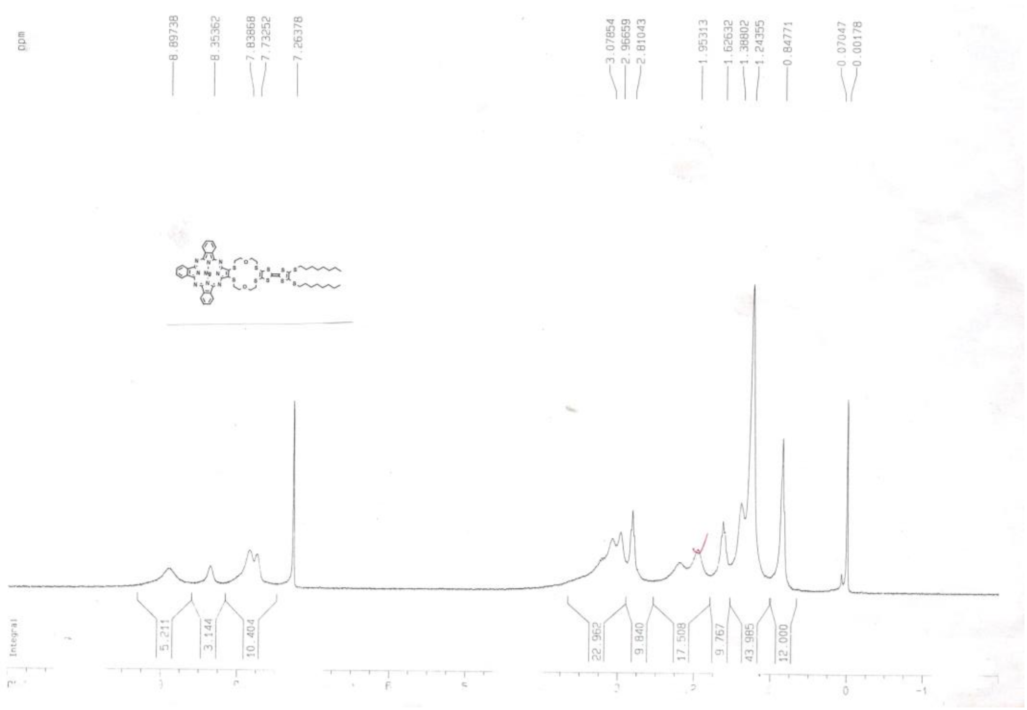

Figure S1. ^{1}H NMR of the target compound **1**

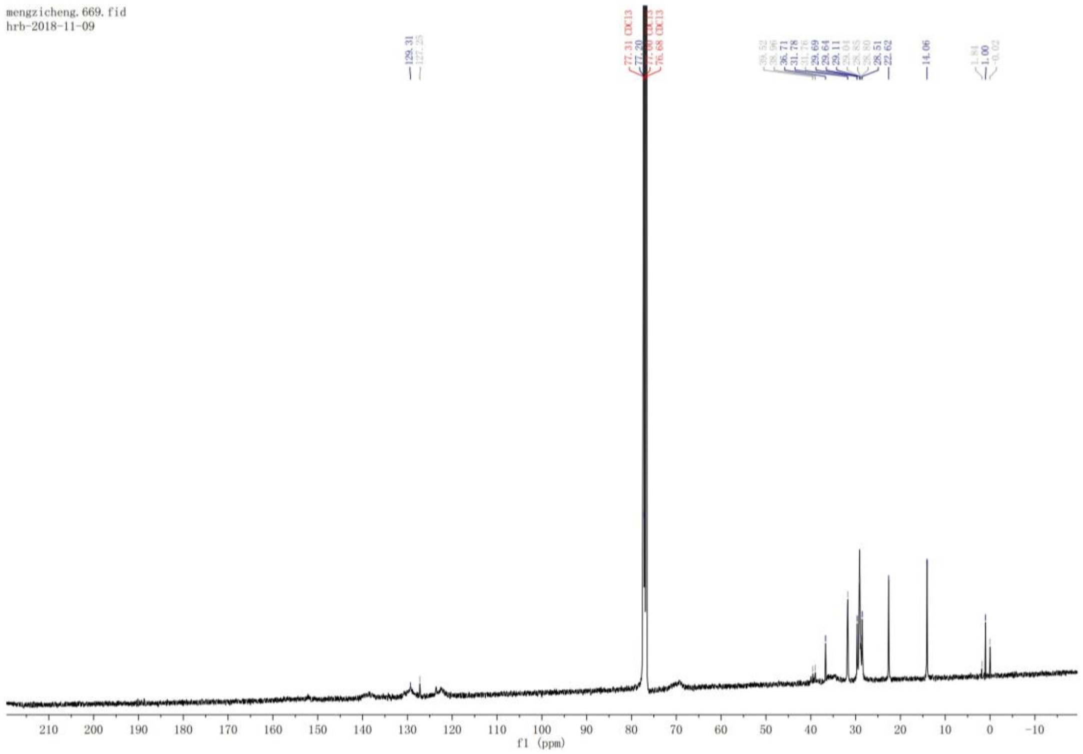

Figure S2. ^{13}C NMR of the target compound **1**

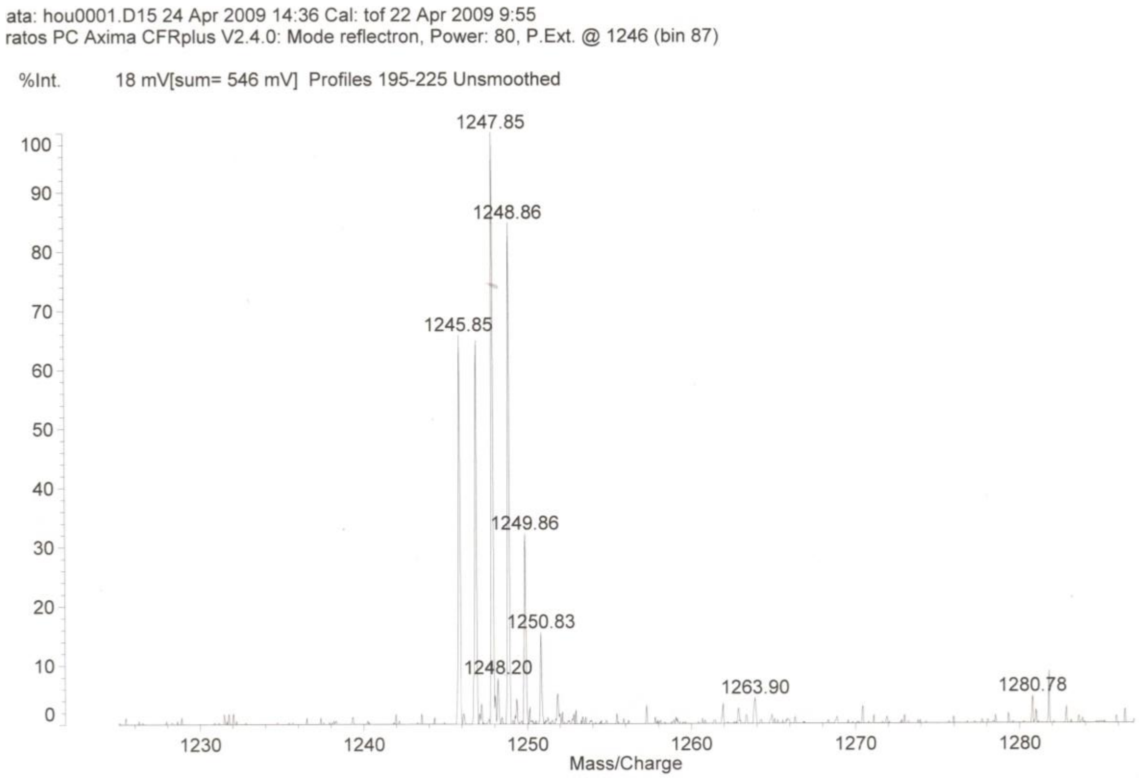

Figure S3. MALDI-TOF-MS of the target compound **1**

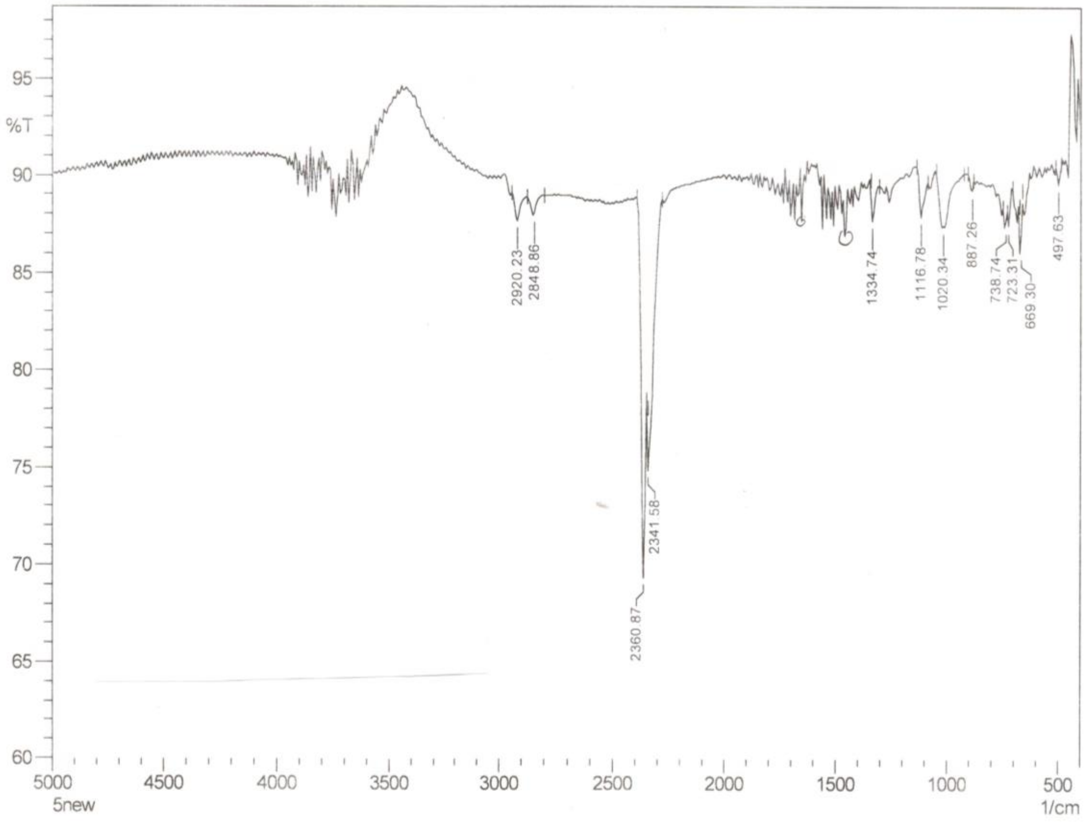

Figure S4. FT-IR of the target compound **1**

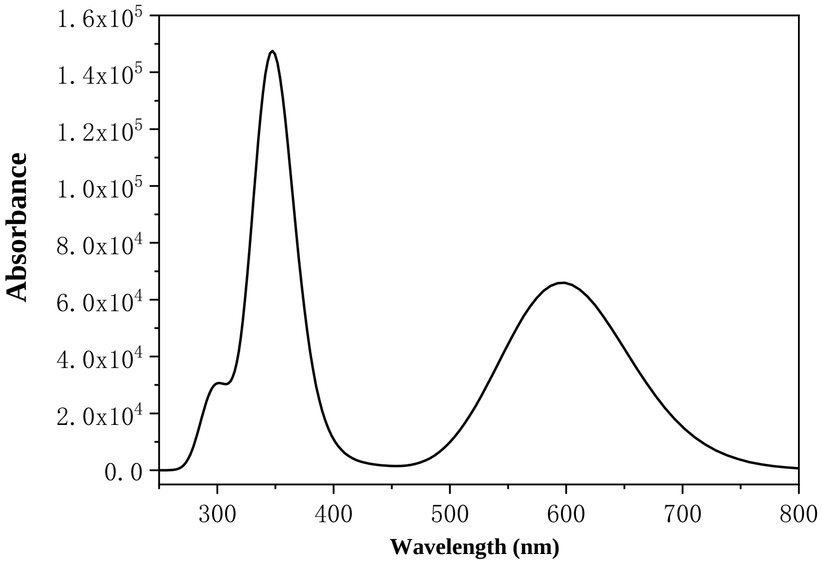

Figure S5. Electron absorption spectrum with B3LYP method computation fitting

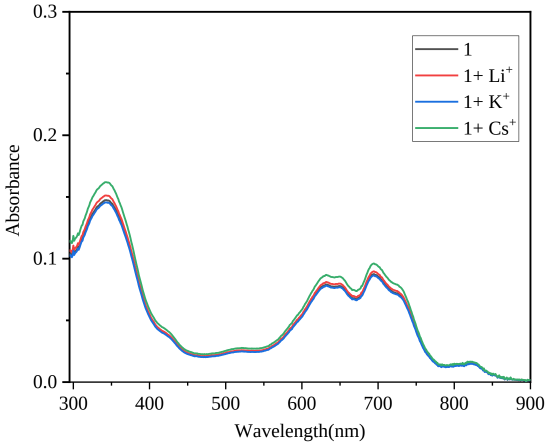

Figure S6. UV–vis titration spectra of Mg-NPc 1 in CH_2Cl_2-MeOH (7:3, v/v) in the presence of different amounts of alkali metal ions.

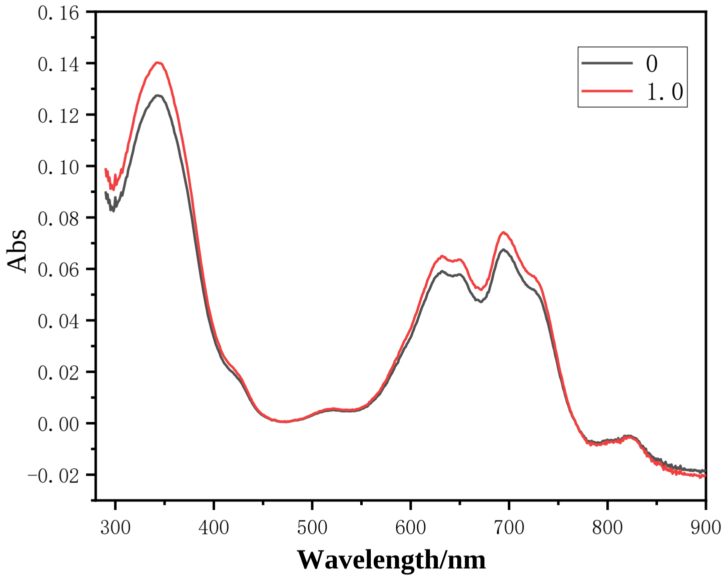

Figure S7. UV–vis titration spectra of Mg-NPc 1 in CH_2Cl_2-MeOH (7:3, v/v) under addition of Zn^{2+}(1.0 equv.)
